# Stress–Dilatancy Behavior of Highly Elastic Rubber-Added Cohesionless Materials

**DOI:** 10.3390/ma17215264

**Published:** 2024-10-29

**Authors:** Haifeng Zhang, Xinrui Zhang, Linjie Li, Zihua Jiang

**Affiliations:** 1 Key Laboratory of Geomechanics and Embankment Engineering of the Ministry of Education, Geotechnical Research Institute, Hohai University, 1 Xikang Road, Nanjing 210024, China; hfzhang1@hhu.edu.cn (H.Z.); linjieli@hhu.edu.cn (L.L.); jiangzihua108@163.com (Z.J.); 2 School of Civil Engineering, Suzhou University of Science and Technology, Suzhou 215009, China

**Keywords:** dilatancy, rubber-added cohesionless materials (RCM), highly elastic, comparative analysis, parameter calibration

## Abstract

Dilatancy is commonly defined as the ratio of the rates of plastic volumetric strain to plastic deviatoric strain, denoted as *D*^p^. Owing to the high modulus of elasticity, the elastic volumetric and deviatoric strain rates under shear stress in conventional cohesionless materials are negligible. Therefore, using the ratio of the rates of total volumetric to deviatoric strain (*D*^t^) as an approximation is common in studying stress–dilatancy behavior and calibrating dilatancy model parameters. This approach is also common in the study of rubber-added cohesionless materials (RCM). However, RCM with a common range of rubber content exhibit a significantly lower modulus of elasticity compared to conventional cohesionless materials. Further research is needed to evaluate the potential impact of elastic strain rates in RCM on stress–dilatancy analysis. Therefore, comparisons were conducted on the stress–dilatancy responses of a series of tests on RCM, where dilatancy is calculated by *D*^p^ and *D*^t^, respectively. Furthermore, a modified method for calibrating the parameters of a state-dependent dilatancy model considering *D*^p^ is presented. It turns out that *D*^p^ is better suited and more precise for dilatancy analysis on highly elastic RCM. Additionally, the dilatancy model can more precisely capture the test results of RCM with parameters calibrated by the proposed method.

## 1. Introduction

The growing abundance of used rubber has brought about widespread attention to its reuse and recycling. Employing it for the creation of rubber-added cohesionless materials (RCM) in geotechnical construction applications is both economically and environmentally friendly, along with contributing towards the establishment of a sustainable society. For example, rubber-cohesionless soil mixtures have been widely and successfully used for lightweight filler of foundations and retaining walls [1,2,3,4,5], seismic isolation and liquefaction potential reduction systems for buildings [6,7,8,9,10,11,12], and as a substitute material for leachate collection systems in landfills [13,14,15]. Additionally, more possible alternative types of RCM, such as rubber–steel furnace slag–coal wash mixtures [16,17,18], rubber–glass mixtures [19,20,21], and rubber–polyurethane urea mixtures [22], have also been suggested for construction applications. However, despite the extensive application potential and prospects of the RCM, statistical data indicates that to date less than 10% of waste rubber has been effectively utilized in construction purpose globally [23]. Therefore, further experimental and theoretical research on RCM remains essential to support and expand the practical applications, aiming to achieve reduced material waste or improved resilience.

Stress–dilatancy is a fundamental aspect of understanding the stress–strain behavior of construction materials [24,25,26]. A typical method for analyzing dilatancy involves formulating a mathematical relationship between the stress ratio, *η*, and the dilatancy, *D*. Dilatancy describes the tendency of volume change in the soil element under shear stress, commonly defined as *D* = d$\varepsilon_{v}^{p}$/d$\varepsilon_{q}^{p}$ [24,27,28]. However, the plastic deformation component cannot be directly measured in triaxial tests; instead, it requires further calculation by subtracting the elastic deformation component from the total deformation. Due to the highly elastic stiffness of conventional cohesionless materials, the elastic deformation component under stress is relatively small. Therefore, when analyzing dilatancy though experimental results and calibrating the parameters for a dilatancy model, it is common practice to ignore the small elastic deformations d$\varepsilon_{v}^{e}$ and d$\varepsilon_{q}^{e}$, and use d*ε_v_*/d*ε_q_* as an approximation [24,29,30,31]. Additionally, there are also studies that first calibrate the elastic modulus for the calculation of elastic deformations, then compute the dilatancy using the equation: d$\varepsilon_{v}^{p}$/d$\varepsilon_{q}^{p}$ = (d*ε_v_* − d$\varepsilon_{v}^{e}$)/(d*ε_q_* − d$\varepsilon_{q}^{e}$) [32,33,34,35]. Specially, they define the total ratio d*ε_v_*/d*ε_q_* as the dilatancy *D*, and define d$\varepsilon_{v}^{p}$/d$\varepsilon_{q}^{p}$ as the plastic dilatancy *D*^p^. Although these studies have subtle differences in the definition, they all emphasize that stress–dilatancy is a concept regarding the ratio of plastic strain rates (d$\varepsilon_{v}^{p}$/d$\varepsilon_{q}^{p}$). Therefore, it can be concluded that the value d$\varepsilon_{v}^{p}$/d$\varepsilon_{q}^{p}$ is crucial in the study of stress–dilatancy. Additionally, it is more preferable to consider total deformation as an approximation (d$\varepsilon_{v}^{p}$/d$\varepsilon_{q}^{p}$ ≈ d*ε_v_*/d*ε_q_*) only when elastic deformation is minimal. In this paper, to avoid misunderstanding, d*ε_v_*/d*ε_q_* is denoted as *D*^t^ and d$\varepsilon_{v}^{p}$/d$\varepsilon_{q}^{p}$ is denoted as *D*^p^. To study the stress–dilatancy mechanical properties of RCM, numerous triaxial tests have been conducted (e.g., [16,17,36,37,38,39,40,41,42,43]). Those studies show that the increased rubber content always increases the shear contraction during hardening of RCM samples, and the dilation after the peak strength decreased. Experimental findings encouraged further research into the dilatancy modelling of RCM. The dilatancy behavior of RCM is similar to that of sands, where dilatancy depends not only on stress state but also on density state. Therefore, the state-dependent dilatancy function introduced by Li and Dafalias [24] has been widely adopted as the basic framework for capturing the dilatancy of RCM in several studies (e.g., [16,36,38,42,44]). Similar to the studies on the conventional cohesionless materials, most of the above experimental or theoretical studies used *D^t^* (d*ε_v_*/d*ε_q_*) to study the stress–dilatancy responses of RCM or calibrated the parameters of the state-dependent dilatancy model. The symbol d$\varepsilon_{v}^{p}$/d$\varepsilon_{q}^{p}$ was defaulted to denote dilatancy in studies [16,38]; however, total deformation was still used for dilatancy analysis or parameter calibration in their research. For instance, they used the extreme value of total volumetric deformation, where *D*^t^ = 0, to determine the phase transformation state. To date, only limited studies have attempted to use the *D*^p^ (d$\varepsilon_{v}^{p}$/d$\varepsilon_{q}^{p}$) for analyzing the stress–dilatancy responses of RCM. Tawk and Indraratna [42] distinguished between the total volumetric strain measured by displacement monitoring devices and the pore volumetric strain measured by the volume of drained water, and represented plastic deformation with pore volumetric strain. Zhang et al. [44] used the data of the residual stage to approximate plastic deformation. Szypcio [34] proposed a method using plastic deformation for the dilatancy analysis of cohesionless sand, which was later applied to rubber–sand mixtures [45]. It can be seen that the analysis of dilatancy in RCM is similar to that in conventional cohesionless materials; it is common to use *D*^t^, and some researchers also attempted to use *D*^p^. In fact, the addition of rubber significantly decreases the elastic stiffness of the RCM [36,46,47].This suggests that the elastic deformation under shear stress of the RCM must increase with the addition of rubber; however, there is a lack of comprehensive and robust quantitative analysis on the difference between *D*^t^ and *D*^p^ considering this impact. Therefore, the potential impact of elastic strain rates in RCM on stress–dilatancy analysis and whether elastic deformation is still minimal needs further study. Additionally, if *D*^p^ is more suitable and precise, modified parameter calibration methods for the state-dependent dilatancy model considering *D*^p^ also need further development. Considering practical applications, the comparative analysis in this study focuses on the common range of optimum rubber content derived from the existing literature (approximately between 20% and 40%) [38,48,49,50,51].

In this paper, the comparative analysis between the *η*–*D^t^* and *η*–*D^p^* curves of several types of RCM with different rubber contents is conducted (within the common range of optimum rubber content). The comparison results indicate that *D^p^* is more suitable for practice analysis in highly elastic RCM. Furthermore, a novel modified method for calibrating state-dependent dilatancy model parameters, especially proposed for the highly elastic RCM, is presented. The effectiveness of the proposed method is evaluated by simulating the *η*–*D*^p^ and *η*–*D*^t^ responses from a series of tests on RCM, and these simulations are also compared with those computed by parameters calibrated by the original method. The results demonstrate that the dilatancy parameters calibrated by the proposed method can more precisely capture the test results under different values of stresses and rubber contents.

## 2. Basic Theoretical Background

Within the generalized triaxial compression tests (*σ*_2_ = *σ*_3_ and *ε*_2_ = *ε*_3_), the stress and strain variables in the *p-q* space can be defined as the mean effective pressure, *p* = (*σ*_1_ + 2*σ*_3_)/3, deviatoric stress, *q* = *σ*_1_ − *σ*_3_, where *σ*_1_ and *σ*_3_ are the major and minor principal stresses, respectively, volumetric strain, *ε_v_* = *ε*_1_ + 2*ε*_3_, and deviatoric strain, ε*_q_* = 2(*ε*_1_ − *ε*_3_)/3, where *ε*_1_ and *ε*_3_ are the major and minor principal compressive strains, respectively.

According to the critical state soil mechanics [28], the total deformation is the sum of the elastic and plastic components, as given by:

(1) $$d\varepsilon_{v}=d\varepsilon_{v}^{e}+d\varepsilon_{v}^{p}$$

(2) $$d\varepsilon_{q}=d\varepsilon_{q}^{e}+d\varepsilon_{q}^{p}$$

where the superscript ‘*e*’ and subscript ‘*p*’ denote the elastic and plastic components of the strain increment, respectively.

The total dilatancy can then be calculated as *D*^t^ *=* d*ε_v/_*d*ε_q_*, and the plastic dilatancy *D*^t^ can then be obtained by combining Equations (1) and (2) as:

(3) $$D^{p}=\frac{d\varepsilon_{v}^{p}}{d\varepsilon_{q}^{p}}=\frac{d\varepsilon_{v}-d\varepsilon_{v}^{e}}{d\varepsilon_{q}-d\varepsilon_{q}^{e}}=\frac{d\varepsilon_{v}-dp/K}{d\varepsilon_{q}-dq/3G}$$

where *K* and *G* represent the bulk modulus and shear modulus of elasticity, respectively.

Equation (3) calculates the ratio of differential incremental rates, which is inherently noisy due to the test error and inhomogeneity in sample preparation. For practical use, the central-difference approach suggested by Been and Jefferies [33] is adopted to reduce such noise, as given by:

(4) $$D_{i}^{p}=\frac{d\varepsilon_{v,i}^{p}}{d\varepsilon_{q,i}^{p}}=\frac{\left(\varepsilon_{v,i+1}-\varepsilon_{v,i-1})-(\varepsilon_{v,i+1}^{e}-\varepsilon_{v,i-1}^{e}\right)}{\left(\varepsilon_{q,i+1}-\varepsilon_{q,i-1})-(\varepsilon_{q,i+1}^{e}-\varepsilon_{q,i-1}^{e}\right)}=\frac{(\varepsilon_{v,i+1}-\varepsilon_{v,i-1})-(p_{i+1}-p_{i-1})/K_{i}}{(\varepsilon_{q,i+1}-\varepsilon_{q,i-1})-(q_{i+1}-q_{i-1})/3G_{i}}$$

where the subscript ‘*i*’ indicates the *i*th incremental step, spanning from the *i* − 1th to the *i* + 1th test points. The elastic bulk modulus *K_i_* and shear modulus *G_i_* are considered constant at the current *i*th incremental step, and only changes when the step moves to the next.

To model the elastic deformation of RCM, the modified function of shear modulus *G* dependent on *p* and *e* by Li and Dafalias [24] has been successfully applied in several studies [36,37,44].

(5) $$G=G_{0}\frac{{\left(2.97-e\right)}^{2}}{1+e}{\left(pp_{a}\right)}^{1/2}$$

where the void ratio *e* is modified as the current value, rather than the initial value *e*_0_ as referenced in [52], and *G*_0_ represents a dimensionless material constant. The shear modulus at the *i*th incremental step can then be expressed as *G_i_* = *G*_0_ (2.97 − *e_i_*)^2^/(1 + *e_i_*) (*p_i_p*_a_)^1/2^. If a complete set of *e*_0_, *p*_0_ (initial mean stress), *ε_v_*_,_*_i_* − *ε_q_*_,_*_i_*, and *ε_q_*_,_*_i_* − *q_i_* data is provided, under a conventional drained stress paths (d*q*/d*p* = 3), *p_i_* and *e_i_* can then be calculated as *p_i_* = *p*_0_ + *q_i_*/3 and *e_i_* = (1 + *e*_0_)/exp(*ε_v_*_,_*_i_*) − 1, respectively. Within the framework of elasticity theory [53], the elastic bulk modulus *K* is equal to:

(6) $$K=G\frac{2(1+\nu )}{3(1-2\nu )}$$

where *ν* is Poisson’s ratio, considered a material constant. The *i*th elastic bulk modulus *K_i_* can then be expressed as *K_i_* = 2/3*G_i_*(1 + *v*)/(1 − 2*v*). Although these elastic parameters were used for calculating the elastic deformations in the constitutive model proposed by Li and Dafalias [24], owing to the high elastic modulus of conventional cohesionless materials, the elastic deformations were neglected when calibrating the dilatancy parameters. Studies in [36,37] also followed this approach. 

## 3. Comparative Analysis on *η*–*D*^t^ and *η*–*D*^p^

The comparison of the *η*–*D*^t^ and *η*–*D*^p^ is evaluated using a series of triaxial drained shear tests conducted on two types of RCM: (i) Rubber–Ayutthaya sand mixtures conducted by Youwai and Bergado [36]. The mixed rubber has the aspect ratio of 1, and the samples were prepared by the under-compaction method. Ayutthaya sand and samples with *R*_c_ varying from 20% to 40% (corresponding to the optimum content range summarized in the introduction section), isotopically compressed at effective confining pressures of 50, 100, and 200 kPa, were used for analysis. (ii) Rubber–beach sand mixtures conducted by Mashiri et al. [37,38]. The mixed rubber has the aspect ratio of 2.8, and the specimens were prepared using the dry deposition method in [54]. Samples with *R*_c_ = 35% (the optimum rubber content specially suggested in their study), isotopically compressed at 23, 69, and 138 kPa, were employed for analysis in this paper. The elastic parameters needed for the calculations follow those calibrated in [36,37], and are summarized in Table 1. The value of *G*_0_ is not directly provided in [37]; instead, the initial shear modulus at pre-shear state (*G*_in_) is given. Therefore, the parameter *G*_0_ in Table 1 of [37] is further calculated using the following equation:

(7) $$G_{0}=G_{in}\frac{1+e_{0}}{{\left(2.97-e_{0}\right)}^{2}{\left(pp_{a}\right)}^{1/2}}$$

where the *G*_in_ denotes initial shear modulus, *e*_0_ denotes the initial void ratio. The above equation is a variation of Equation (5).

### 3.1. Negligible Elastic Deformation in the Dilatancy of Conventional Cohesionless Materials

**Figure 1:**
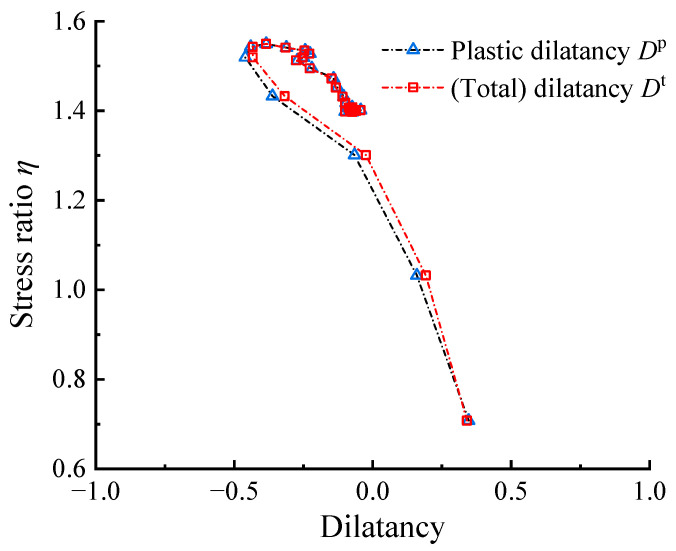
Comparison of the *η*–*D*^p^ and the *η*–*D*^t^ curves of Ayutthaya sand with *p*_0_ = 100 kPa (*ε_v_*_,*i*_–*ε_q_*_,*i*_ and *ε_q_*_,*i*_–*q_i_* data set needed for calculating the *η*–*D* responses were obtained from Youwai and Bergado [36] using GetData Graph Digitizer 2.22).

Figure 1 shows the *η*–*D*^t^ and the *η*–*D*^p^ curves calculated from the drained shear tests on Ayutthaya sand with confining stress *p*_0_ = 100 kPa (the host sand used in Youwai and Bergado [36] for RCM specimen preparation). The *η*–*D*^t^ curve is very close to the *η–D^p^* curve. This result is consistent with the generally accepted assumption that the small elastic deformations of conventional cohesionless materials can be ignored for dilatancy analysis, and the use of *η*–*D*^t^ is an approximation.

### 3.2. Impact of Elastic Deformation in the Dilatancy of Highly Elastic Cohesionless Materials

**Figure 2:**
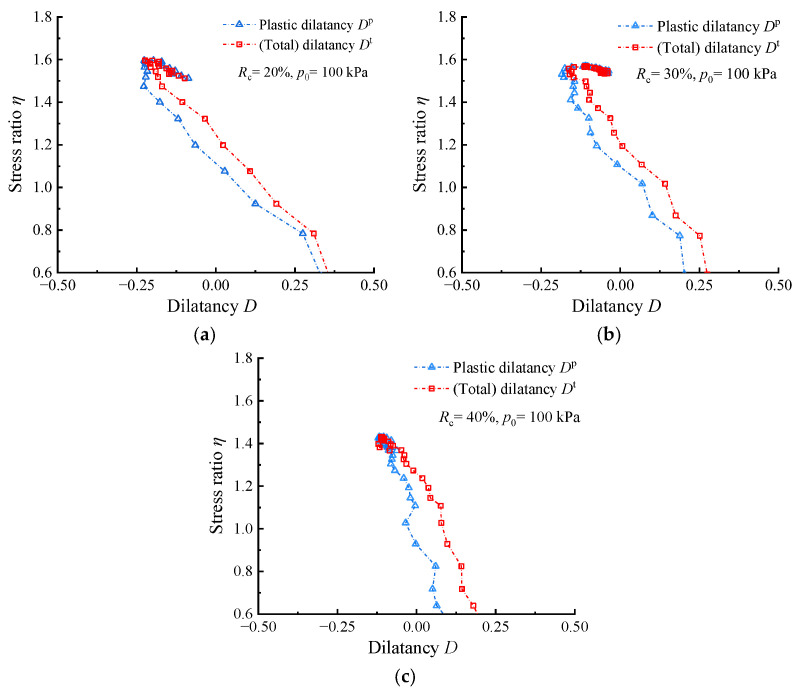
Comparison of *η*–*D*^p^ and *η*–*D*^t^ curves for RCM at different *R*_c_ values with *p*_0_ = 100 kPa: (**a**) *R*_c_ = 20%; (**b**) *R*_c_ = 30%; (**c**) *R*_c_ = 40% (*ε_v_*_,*i*_–*ε_q_*_,*i*_ and *ε_q_*_,*i*_–*q_i_* data set needed for calculating the *η*–*D* responses were obtained from Youwai and Bergado [36] using GetData Graph Digitizer 2.22).

The impact of the highly elastic characters on dilatancy analysis was initially assessed using the test data from drained shear tests on rubber–sand mixtures conducted by Youwai and Bergado [36] with a confining pressure *p*_0_ of 100 kPa. Figure 2a shows that once the *R*_c_ reaches 20%, a noticeable gap emerges between the two curves in stress hardening. Furthermore, as illustrated in Figure 2b,c, the aforementioned gap becomes more pronounced with the increase in *R*_c_.

**Figure 3:**
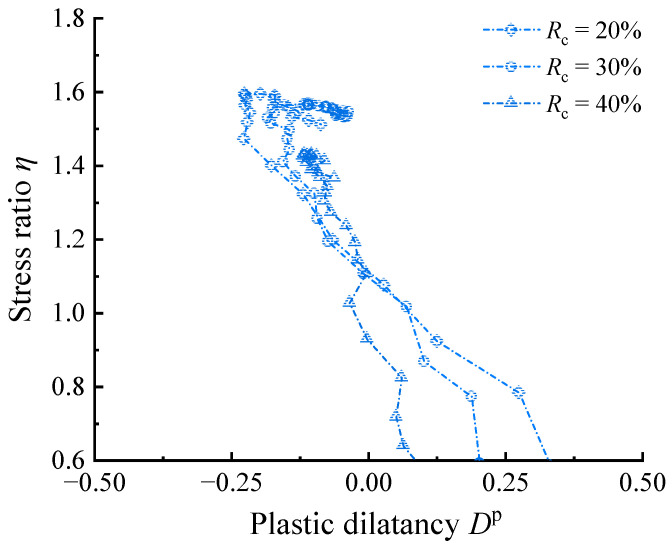
Comparison of *η*–*D*^p^ curves for RCM at different *R*_c_ values with *p*_0_ = 100 kPa.

The specific effects of using *η*–*D* instead of *η*–*D*^p^ for dilatancy analysis need further discussion. Figure 2 shows that the *η*–*D* curves in the strength hardening (d*η* > 0) is generally located on the right side of the *η*–*D*^p^ curves (*D*^t^ > *D*^p^, at the same *η*), which indicates that the high elastic deformation of the RCM makes a great contribution to the higher shear volume shrinkage and smaller shear volume expansion in the shear test. Therefore, ignoring the elastic deformation will exaggerate the resistance to shear expansion in RCM. In addition, Figure 2 shows that the phase transformation (dilatancy) stress ratio *M*_d_ calibrated by *η*–*D*^t^ overestimates the actual value relative to that calibrated by *η*–*D*^p^. This is because the existence of elastic volume contraction deformation offsets the part of the plastic volume dilatation deformation, thus the state that *D*^t^ = 0 is later than *D*^p^ = 0; since the samples are in the hardening stage, the later points have higher stress ratios. Therefore, within the common optimum range of rubber content, *η*–*D*^p^ is more recommended for analyzing stress–dilatancy for RCM. Figure 3 compares the *η*–*D*^p^ curves of RCM at different *R*_c_ values. It can be observed that as the *R*_c_ increases, the absolute value of *D*^p^ decreases, indicating that both plastic contraction and dilation are diminishing.

**Figure 4:**
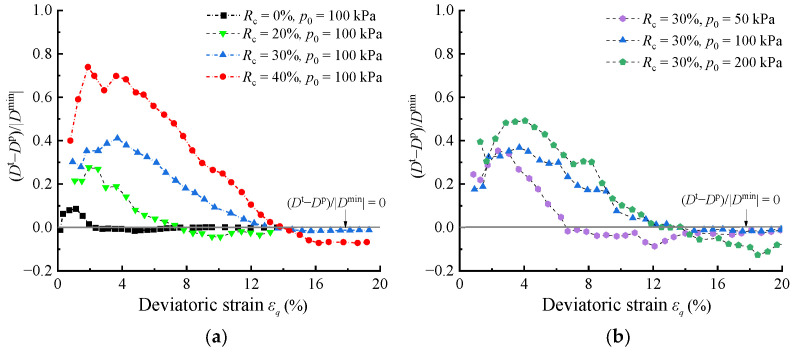
(*D*^t^ − *D*^p^)/|$D_{min}^{p}$| under different triaxial test conditions: (**a**) different *R*_c_; (**b**) different *p*_0_.

The previous analysis provided a general overview. To further quantify the estimated error arising from replacing *D*^p^ with *D*^t^ in shear, the modified relative error: (*D*^t^ − *D*^p^)/|$D_{min}^{p}$| versus *ε_q_* under varying *p*_0_ and *R*_c_ conditions are further analyzed. Here, *D*^p^ was replaced with |$D_{min}^{p}$| to prevent the denominator from being zero. Overall, Figure 4a,b depict that (*D*^t^ − *D*^p^)/|$D_{min}^{p}$| values increase with the increase in *R*_c_ and *p*_0_, and the influence of *R*_c_ is much more significant. Notably, when the curves in Figure 4 intersect the *x*-axis, samples reach their peak stress state, characterized by d*q* = d*p* = 0. According to Equation (3), *D*^t^ = *D*^p^ and (*D*^t^ − *D*^p^)/|$D_{min}^{p}$| = 0 can be derived when d*q* = d*p* = 0. Using the peak stress state as a reference point, the region above the *x*-axis corresponds to the stress hardening, while the region under the *x*-axis corresponds to the stress softening. During the stress hardening, the modified relative error of all tests first increases and then decreases to 0 at the peak stress state. During stress softening, the modified relative error of host sand (*R*_c_ = 0) is almost zero. However, the error of RCM in stress softening gradually increases with increasing *p*_0_ and *R*_c_, but it is still much smaller than that in stress hardening. The maximum (*D*^t^ − *D*^p^)/|$D_{min}^{p}$| value in RCM with *R*_c_ = 40% approaches eight times greater than that with *R*_c_ = 0%.

**Figure 5:**
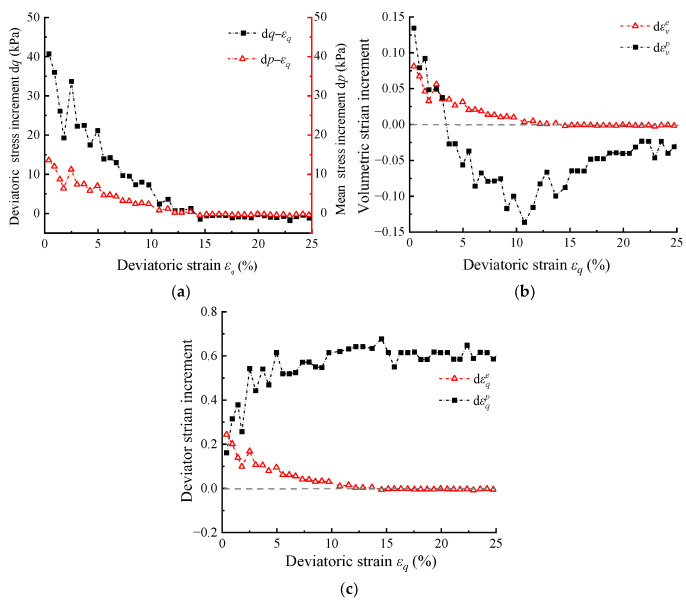
The relationships of stresses and strain increments with deviatoric strain: (**a**) d*q* and d*p* versus *ε_q_*; (**b**) d$\varepsilon_{v}^{e}$ and d$\varepsilon_{v}^{p}$ versus *ε_q_*; (**c**) d$\varepsilon_{q}^{e}$ and d$\varepsilon_{q}^{p}$ versus *ε_q_* (taking the test condition with *R*_c_ = 30% and *p*_0_ = 100 kPa in [36] as an example).

This phenomenon can be explained using Figure 5 (taking the test condition with *R*_c_ = 30% and *p*_0_ = 100 kPa as an example). As shown in Figure 5a, during stress hardening (d*q* > 0), the stress increments d*p* and d*q* gradually decrease with the increase of *ε_q_*, and finally decrease to 0 at the peak stress ratio. These values then continue to decrease to negative values during stress softening (d*q* < 0), but the magnitude of the change is much lower than that during hardening. Based on Equation (3), it can be observed that the magnitudes of d*p* and d*q* determine the magnitudes of d$\varepsilon_{v}^{e}$ and d$\varepsilon_{q}^{e}$. As shown in Figure 5b,c, during stress hardening, the decreasing values of d*p* and d*q* lead to a gradual reduction in d$\varepsilon_{v}^{e}$ and d$\varepsilon_{q}^{e}$, resulting in an overall decreasing trend in modified relative error. The temporary increase in relative error during the early stage of hardening is caused by the temporary convergence of the d$\varepsilon_{v}^{p}$ and d$\varepsilon_{v}^{e}$ shown in Figure 5b, which increases the relative proportion of elastic volumetric increment and leads to a transient increase in the modified relative error. After reaching the peak stress state, d*p* and d*q* decrease to zero, resulting in zero values of modified relative error. During stress softening, d*p* and d*q* continue to decrease as negative values, but their magnitudes are smaller compared to that in stress hardening. As a result, the negative elastic deformation generated is also smaller, leading to a much smaller relative error.

**Figure 6:**
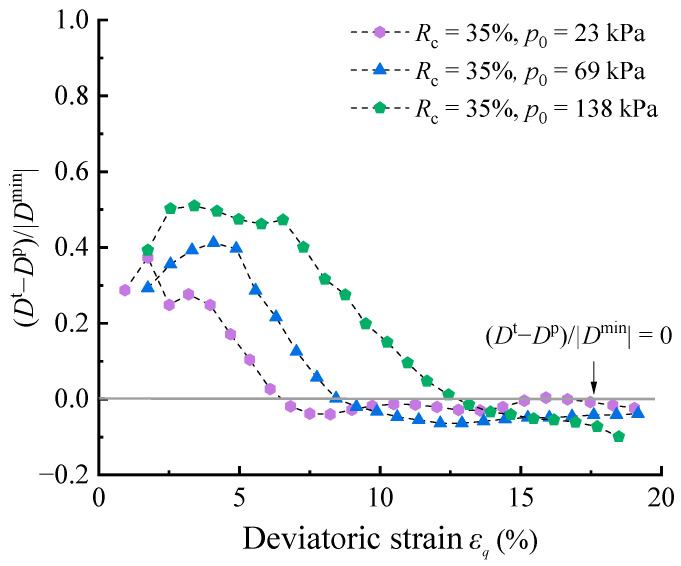
(*D*^t^ − *D*^p^)/|$D_{min}^{p}$| under different *R*_c_ conditions (data set needed for calculation were obtained from Mashiri et al. [38] using GetData digitization software).

The impact of the elastic deformations in the analysis of dilatancy was further quantified using the (*D*^t^ − *D*^p^)/|$D_{min}^{p}$| results calculated from drained shear test data of the RCM in [37,38] with *R*_c_ = 35%. As shown in Figure 6, the experimental characteristics of the aforementioned analysis are reproducible and the errors are also at a relatively high level, which confirms the reliability of the previous conclusions.

## 4. Calibration of Dilatancy Parameters Considering Plastic

The RCM exhibits state-dependent dilatancy characteristics similar to conventional granular materials. Specifically, for the same value of *η*, loose specimens exhibit contraction while dense specimens exhibit dilation. Therefore, the test results of RCM have been analyzed using the state-dependent dilatancy function introduced by Li and Dafalias [24], as demonstrated in several studies (e.g., [16,17,36,39,42,44]).

(8) $$D^{p}=D_{0}(e^{m\psi }-\frac{\eta }{M})$$

where e represents Euler’s number, *D*_0_ and *m* are model parameters, and *ψ* = *e* − *e*_cs_ is the state parameter as defined by Been and Jefferies [55]. This parameter measures the deviation of the soil state, given the current void ratio *e* and confining pressure *p*, from the critical state. The empirical formula *e*_cs_ = *e_Γ_* − *λ*_c_(ln*p*) is used instead of *e*_cs_ = *e_Γ_* − *λ*_c_(*p*/*p*_a_)*^ξ^*, which was used in [24] to depict the critical state line of RCM in their studies, where *e_Γ_*, *λ*_c_ are the critical state constants.

### 4.1. Calibration Methods for Sand

The state-dependent model was originally proposed for sand. Therefore, parameters *m* and *D*_0_ can be determined by neglecting the small elastic deformation of the sand in shear tests [24]. Hence:

(9) $$D^{t}=\frac{d\varepsilon_{v}}{d\varepsilon_{q}}\approx D^{p}=\frac{d\varepsilon_{v}^{p}}{d\varepsilon_{q}^{p}}=D_{0}(e^{m\psi }-\frac{\eta }{M})$$

For drained triaxial tests, the constant *m* can then be calibrated by Equation (9) at *D*^t^ = 0 (peak of the volumetric contraction strain [31]). Hence:

(10) $$m=\frac{1}{\psi_{d}}\ln(\frac{M_{d}}{M})$$

where *M*_d_ is the transformation stress ratio determined at *D*^t^ = 0; *ψ_d_* is the value of *ψ* at *D*^t^ = 0.

After *m* is determined, *D*_0_ can be subsequently determined by fitting the measured *ε_q_*–*ε_v_* curves using Equation (9) [24,31].

### 4.2. Calibration Methods for Highly Elastic Materials

#### 4.2.1. Calibration of the Parameter *m*

**Figure 7:**
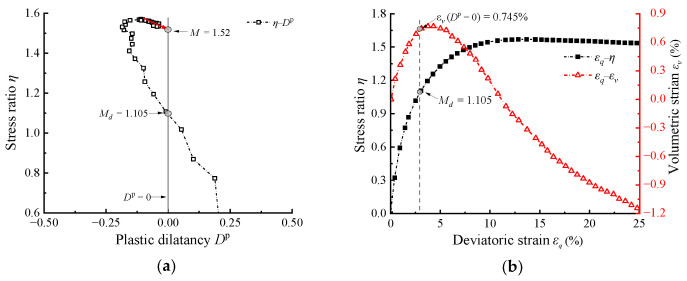
Method for calibrating the parameter *m* of highly elastic RCM: (**a**) determination of the *M*_d_; (**b**) determination of the extreme value of plastic volumetric strain.

As previously mentioned, *m* is calculated using *M*_d_ and *ψ*_d_ at the phase transformation state. For highly elastic RBM, previous studies have used various descriptions to define this state; e.g., the state where the *D*^t^ = d*ε_v_*/d*ε_q_* = 0 [36], the state where the *ε_v_* reaches its minimum value [16,38,39]. In summary, these descriptions collectively indicate the location of maximum total compressive volumetric deformation (*D*^t^ = 0), which corresponds to the densest state under shear. However, the previous section of this paper has shown that using *D^t^* = 0 will delay the phase transformation state, and overestimate *M*_d_. Therefore, a modified calibration method for *m* is proposed. As shown in Figure 7a, a more precise phase transformation stress *M*_d_ is determined based on the *η*–*D*^p^ curve. Then, the deviatoric strain at the phase transformation state *ε_q_* (*η* = *M*_d_) of the materials is determined according to the *ε_q_–η* curve, as shown in Figure 7b. Subsequently, *ε_v_* (*η* = *M*_d_) can be determined based on the *ε_q_–ε_v_* curve for converting the void ratio *e* (*η* = *M*_d_) and calculating the *ψ*_d_. Finally, substituting *M*_d_ and *ψ*_d_ determined by new method into Equation (10) yields a more precise value of *m*. The critical state ratio *M* needed for calculation in Equation (10) is determined by the extrapolation method introduced in [16,56,57]. Specifically, as indicated by the red arrow in Figure 7a, this involves linearly fitting the data near the end of the test and extrapolating it to the point where *D*^p^ = 0.

#### 4.2.2. Calibration of the Parameter *D*_0_

For conventional cohesionless materials, the small elastic deformations are ignored, and parameter *D*_0_ can be determined using Equation (9). For high elastic RCM, elastic deformation can further be considered based on Equation (9). Therefore,

(11) $$D^{t}=\frac{d\varepsilon_{v}}{d\varepsilon_{q}}=\frac{d\varepsilon_{v}^{p}+d\varepsilon_{v}^{e}}{d\varepsilon_{q}^{p}+d\varepsilon_{q}^{e}}=\frac{D_{0}(e^{m\psi }-\frac{\eta }{M})d\varepsilon_{q}^{p}+d\varepsilon_{v}^{e}}{d\varepsilon_{q}^{p}+d\varepsilon_{q}^{e}}$$

The parameter *D*_0_ can be subsequently determined by fitting the measured *ε_q−_ε_v_* curves using Equation (11). As shown in Figure 4, the modified relative error reaches zero at the peak strength states, indicating that there are no influences from elastic deformations. Therefore, it is also feasible to calibrate *D*_0_ using data at peak strength states, as introduced in [16,38].

## 5. Validation of the Parameter Calibration Methods

**Figure 8:**
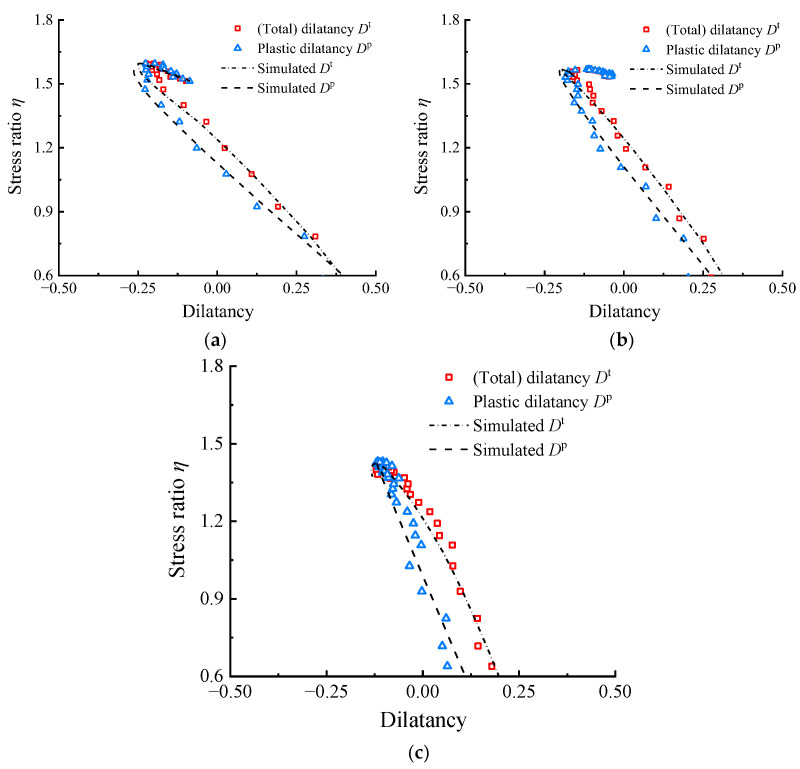
Comparison of the test data and simulation results for RCM at *p*_0_ = 100 kPa with varying *R*_c_: (**a**) *R*_c_ = 20%; (**b**) *R*_c_ = 30%; (**c**) *R*_c_ = 40%.

**Figure 9:**
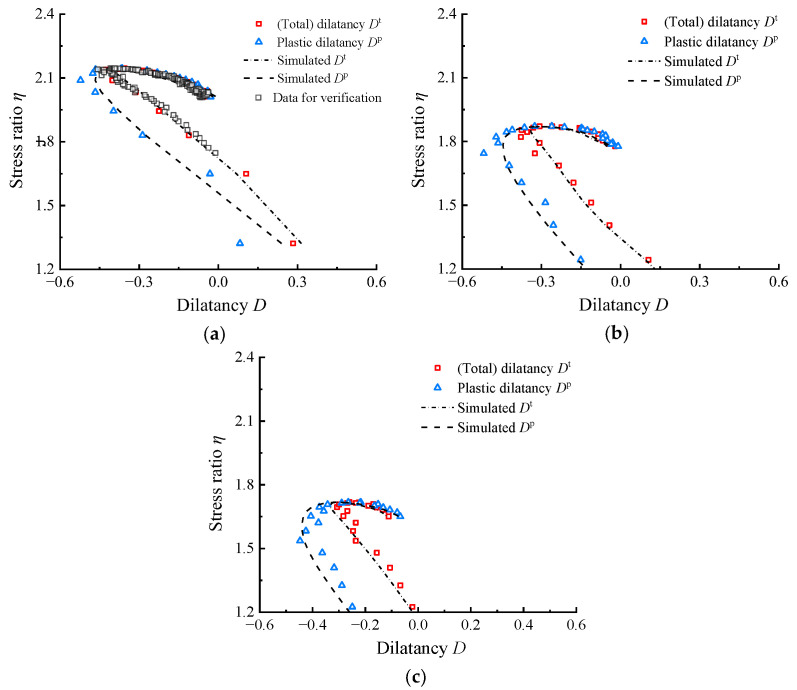
Comparison of the test data and simulation results for RCM at *R*_c_ = 35% with varying *p*_0_: (**a**) *p*_0_ = 23 kPa; (**b**) *p*_0_ = 69 kPa; (**c**) *p*_0_ = 138 kPa (=*ε_v_*_,*i*_–*ε_q_*_,*i*_ and *ε_q_*_,*i*_–*q_i_* data set needed for calculating the *η*–*D* responses were obtained from Mashiri et al. [37,38] using GetData Graph Digitizer 2.22).

The capability of the parameter calibration method was validated using the experimental results from shear tests conducted by Youwai and Bergado [36] and Mashiri et al. [37,38]. All parameters needed for simulation are listed in Table 1 and Table 2. The elastic and critical state parameters follow the values in [36,37], the dilatancy parameters *D*_0_ and *m* are calibrated by the novel modified methods in this paper, and the critical state ratio *M* is determined by extrapolation method introduced in [16,56,57]. Figure 8 and Figure 9 compare the simulated *η*–*D^t^* and *η*–*D^p^* results with the experimental results from [36,38] at different *R*_c_ and *p*_0_ values, respectively. Both the *η*–*D^t^* and *η*–*D^p^* responses simulated using the parameters in this paper can capture the overall trend of the test results of RCM. This demonstrates the ability of model to predict the stress–dilatancy mechanical behavior of highly elastic RCM in a wide range of *R*_c_ and *p*_0_. The stress–dilatancy curves in this paper are calculated based on the limited *ε_v_*_,*i*_–*ε_q_*_,*i*_ and *ε_q_*_,*i*_–*q_i_* data set shown in the literature using GetData. To verify the reliability of the calculation, Figure 9a also compares the *η*–*D^t^* curve represented by gray hollow squares in references [37,38] (The study in [37] indicates that the ratio of the rates of total deformations was used for dilatancy calculation), which was calculated and provided based on abundant original experimental data. Additionally, the positive and negative definitions of dilatancy in this paper are different to theirs, and a negative sign was added for comparison. It can be observed that, although the data points calculated in this paper are relatively few, they match the original experimental data very well, thus confirming the reliability of the calculation method used in this paper.

**Figure 10:**
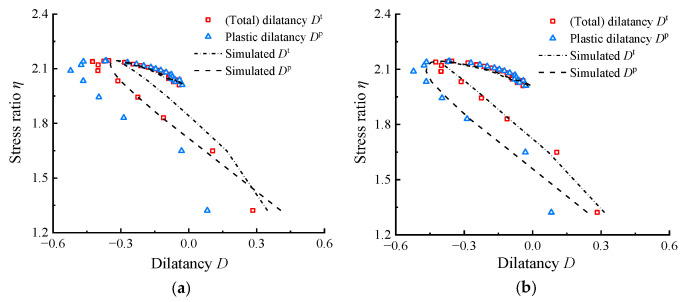
Comparison of the simulation results obtained by two sets of dilatancy parameters: (**a**) simulations using parameters determined by original method; (**b**) simulations using parameters determined by modified method in this paper (using the test condition with *R*_c_ = 35% and *p*_0_ = 23 kPa in [37,38] as an example).

Figure 10 compares the simulation results obtained using the dilatancy parameters calibrated by the proposed method in Table 2 with those obtained using parameters calibrated by the original method. The comparison uses the test conditions with *R*_c_ = 35% and *p*_0_ = 23 kPa from [37,38] as an example, where *D*_0_ = 2.4 and *m* = 3.1 were determined by original method and extrapolated *M*. In Figure 10a, the simulated *η*–*D^t^* and *η*–*D^p^* curves using the parameters calibrated in this paper both exhibit good agreement with tested curves. In Figure 10b, due to the neglect of elastic deformation during parameter calibration, the simulated *η*–*D^p^* matches the measured *η*–*D^t^*, while the simulated *η*–*D^t^* overestimates the overall contraction observed in the measured data.

## 6. Discussion

A series of of *η*–*D^p^* and *η*–*D^t^* curves of RCM are compared, the errors caused by using *η*–*D^t^* instead of *η*–*D^p^* are quantitatively analyzed, and a novel modified method for calibrating the parameters of the state-dependent dilatancy model based on *D^p^* is proposed. The method in this study can more precisely capture the stress–dilatancy results of highly elastic rubber-added materials compared with previous studies. However, for practical purposes, this article focuses solely on tests conducted within the commonly recommended optimal range of rubber gravity content (20–40%). In fact, there should be a smaller range of rubber content that still meets high elastic stiffness to the extent that elastic deformation can be ignored. Therefore, some experiments conducted on RCM with lower rubber content can still be further studied to enrich the possible application range of conclusions in future. In addition, RSM with different rubber–sand particle size ratios and different shape parameters should also be taken into account in the future.

## 7. Conclusions

To analyze the influence of highly elastic properties on the stress*–*dilatancy behavior of RCM, a comparative analysis between *η*–*D*^t^ and *η*–*D*^p^ is conducted, and a novel modified dilatancy model parameter calibration method specifically for these highly elastic rubber-added materials is proposed. The following main conclusions can be drawn:

(1) Compared to *η*–*D*^p^, the *η*–*D*^t^ response tends to overestimate both the phase transformation stress ratio and the reduction capacity in dilatancy for highly elastic RCM. Therefore, the *η*–*D*^p^ response is more suitable for the dilatancy analysis of RCM.
(2) The difference between *D*^p^ and *D*^t^ increases initially with increasing deviatoric strain during the stress hardening, and subsequently decreases, eventually approaching zero at the peak stress state. During stress softening, *D*^p^ and *D*^t^ start to exhibit a difference again, but relatively smaller compared to the stress hardening. In addition, with increasing confining pressure and rubber content, the difference between *D*^p^ and *D*^t^ becomes more significant.
(3) The dilatancy parameters *D*_0_ and *m*, calibrated by the modified method in this paper, can more precisely capture the strength–dilatancy behavior of highly elastic materials under different initial confining stresses and rubber contents.

## Figures and Tables

**Table 1:** Summary of testing conditions and elastic parameters.

Data Source	*R*_c_ (%)	*p*_0_ (kPa)	*e*_0_	*G*_0_	*v*
Youwai and Bergado [36]	20%	100	0.36	18	0.33
	30%	100	0.29	10	0.33
	40%	100	0.30	6	0.33
Mashiri et al. [37,38]	35%	23	0.352	10	0.33
	35%	69	0.347	7	0.37
	35%	138	0.339	5	0.4

**Table 2:** Summary of critical state and dilatancy parameters at different testing conditions.

Data Source	*R*_c_ (%)	*p*_0_ (kPa)	*e*_0_	*M*	*e_Γ_*	*λ*	*m*	*D*_0_
Youwai and Bergado [36]	20%	100	0.36	1.45	0.44	0.0102	8	1.1
	30%	100	0.29	1.45	0.53	0.0302	4	0.8
	40%	100	0.30	1.45	0.66	0.0302	1.5	0.4
Mashiri et al. [37,38]	35%	23	0.352	1.97	0.432	0.01	5	2.1
	35%	69	0.347	1.72	0.432	0.01	9	1.2
	35%	138	0.339	1.61	0.432	0.01	9.8	1.05

## Data Availability

The original contributions presented in the study are included in the article; further inquiries can be directed to the corresponding author/s.
